# Attentional demands during walking are increased by small simulated leg length discrepancy

**DOI:** 10.3389/fnrgo.2025.1629128

**Published:** 2025-12-08

**Authors:** Keisuke Takada, Miyu Sugimoto, Yuma Takenaka, Kenichi Sugawara, Tomotaka Suzuki

**Affiliations:** 1 School of Rehabilitation, Kanagawa University of Human Services, Yokosuka, Japan; 2 Graduate Course of Health and Social Services, Kanagawa University of Human Services, Yokosuka, Japan

**Keywords:** leg length discrepancy, attentional demands, walking, gait asymmetry, reaction time

## Abstract

**Introduction:**

Leg length discrepancy (LLD) is known to disrupt gait symmetry and affect motor control. However, the effects of LLD-induced gait asymmetry on attention functions during walking remain unclear. Therefore, this study aimed to investigate the impact of simulated LLD and walking track on attentional demands and gait parameters in young, healthy adults.

**Methods:**

This prospective study included participants who completed walking trials on straight (*n* = 14) and circular (*n* = 16) tracks under randomly assigned LLD conditions (no lift and 10-, 20-, 30-, and 40-mm shoe lifts). Attentional demands during walking were assessed using a simple reaction time (RT) paradigm. Gait symmetry was evaluated by step-time ratio and triaxial trunk acceleration root mean square (RMS) ratios, calculated from timing and accelerometer data. The data were analyzed using a two-way mixed analysis of variance.

**Results:**

LLD significantly increased RT and step-time ratio compared to zero LLD. However, the circular walking track did not significantly affect RT or step-time ratio. LLD also significantly increased trunk movement asymmetry (RMS ratios). No significant interaction effects were found for all variables.

**Conclusion:**

Simulated LLD significantly increased attentional demands and gait asymmetry, although the rise in attentional demands was limited in healthy participants. The circular walking track had minimal effects and did not exacerbate the challenges associated with LLD. These results provide insights into the effects of gait asymmetry caused by the degree of LLD and walking environment on human gait strategy and its associated attentional demands.

## 1 Introduction

As many as 80% of individuals have a leg length discrepancy (LLD) of < 10 mm (Gordon and Davis, 2019). LLDs are associated with various musculoskeletal disorders, such as low back pain, lower extremity pain, and osteoarthritis. LLDs of >20 mm are generally considered clinically problematic (Gordon and Davis, 2019; Gurney, 2002). However, emerging evidence suggests that even mild LLDs of < 20 mm can cause degenerative changes in the hip joint and lumbar spine (Murray et al., 2017). Furthermore, shoe lifts are effective in treating patients with an LLD of ≤ 10 mm and chronic low back pain (Defrin et al., 2005).

Several studies examining the kinesiological effect of LLDs on gait have revealed an association between moderate LLDs of >20 mm and asymmetrical leg movement (Gurney et al., 2001; Kaufman et al., 1996; Liu et al., 1998; Perttunen et al., 2004). Whether simulated or actual, mild LLDs do not cause asymmetry in lower limb joint moments due to the body's compensatory control (Goel et al., 1997). However, Khamis and Carmeli (2018) reported that 5-mm LLD results in asymmetric changes in dynamic leg length using three-dimensional motion analysis. Therefore, considering the impact of such LLDs on physical functions is important since they do not always adversely affect gait. LLD-induced gait asymmetry is controlled by functional compensations through movements of the pelvis, knee, and ankle (Walsh et al., 2000). Asymmetry of the left and right legs when walking can be easily generated using curved or sloped walking tracks, although asymmetry based on neuromusculoskeletal impairments can impair long-term gait efficiency and stability. In clinical practice, the cause of LLDs and their effects on gait should be properly evaluated. Therefore, the treatment strategy for LLD should be considered for each patient (Vogt et al., 2020).

Although human gait is highly automated, attentional demands increase when walking on a stable, straight track compared to that for a baseline seated condition (Suzuki et al., 2016) and further rise when postural control during walking becomes more difficult or when cognitive tasks are necessary (Suzuki et al., 2019). Since human attention is limited, the risk of falls is increased when simultaneous tasks require divided attention (Yogev-Seligmann et al., 2008). Woollacott and Shumway-Cook (2002) reported that older adults with impaired balance require higher attentional demand for postural control and may be at increased risk of falling when performing multiple tasks that involve postural control. Diminished attention function is among the risk factors associated with a risk of falling (Holtzer et al., 2007). Specifically, increased attentional demands during walking, resulting from impaired motor function and/or decreased attentional capacity due to impaired cognitive function, may lead to delayed Reaction Times (RTs). This makes stepping over obstacles difficult and increases the risk of falls in older adults (Chen et al., 1996). The effects of increased attentional demands on human movement can be studied by manipulating the qualitative and quantitative factors of task difficulty and concurrent tasks respectively, (Suzuki et al., 2019).

Humans use their feet to sensitively monitor minor changes in their shoes and the ground, providing sensory feedback to the central nervous system. These afferent inputs are integrated with other sensory information for subsequent motor control. Therefore, even slight asymmetries, such as the discomfort associated with LLD, can affect gait control and impose a burden on attention functions. This increase in attentional demands can be attributed to the difficulty of postural control caused by gait asymmetry, such as that induced by LLD. During stable walking, the trunk functions primarily to maintain posture. However, when lower-limb stability is compromised, a compensatory postural control strategy involving the trunk, primarily centered around the hip joint, becomes necessary to maintain balance (Horak, 2006). Such compensatory control is thought to impose an additional load on the automated walking process and to further increase attentional demands.

Not much symmetry can be maintained while walking in daily life, particularly when navigating circular paths (Orendurff et al., 2006), which are movements associated with a high risk of falling. Sufficient motor and cognitive functions are required to control different movements for the left and right legs. However, how gait asymmetry induced by LLDs or a walking track affects attention functions and how they interact is unclear.

Although recent high-precision studies suggest that local biomechanical changes, such as kinematic asymmetry or alterations in foot pressure distribution, occur even with very small LLDs of 5 mm (Khamis and Carmeli, 2018; Pereiro-Buceta et al., 2021), the magnitude of LLD at which the compensatory control begins to require significant attentional demands remains unknown. We based our study on the premise that a significant increase in attentional demands occurs when the overall gait pattern is altered, necessitating more conscious control. Therefore, this study aimed to explore this potential threshold by investigating effects of asymmetry, created using a gradually increased LLD (from 10 to 40 mm), on attentional demands while walking on different walking tracks. We hypothesize that corresponding attentional demands will be necessary if gait asymmetry increases to a significant degree.

## 2 Materials and methods

### 2.1 Participants

This prospective study was approved by the Research Ethics Committee of Kanagawa University of Human Services (approval number: 29-7) and conducted according to the principles of the Declaration of Helsinki. The sample size was calculated with reference to our previous study that measured RT during walking (Suzuki et al., 2019) using G^*^Power version 3.1.9.7 (Düsseldorf, Germany). Assuming the use of a paired *t*-test with Bonferroni correction for the RT data, the α, power, and effect size were set to 0.0125, 0.80, and 1.38, respectively. Therefore, the total sample size calculated was 10. In total, 14 (six men and eight women, aged 20–22 years) and 16 (six men and 10 women, aged 19–22 years) participants were included in the straight and circular walking experiments, respectively. Participants were confirmed via interview to have no history of orthopedic diseases, including structural LLD, or neuromuscular problems. Furthermore, a physical therapist screened for obvious asymmetries in gait and posture in the frontal plane that could affect task performance. All participants provided written informed consent prior to the initiation of the study; all data were collected within the facilities of Kanagawa University of Human Services.

### 2.2 Materials

**Figure 1:**
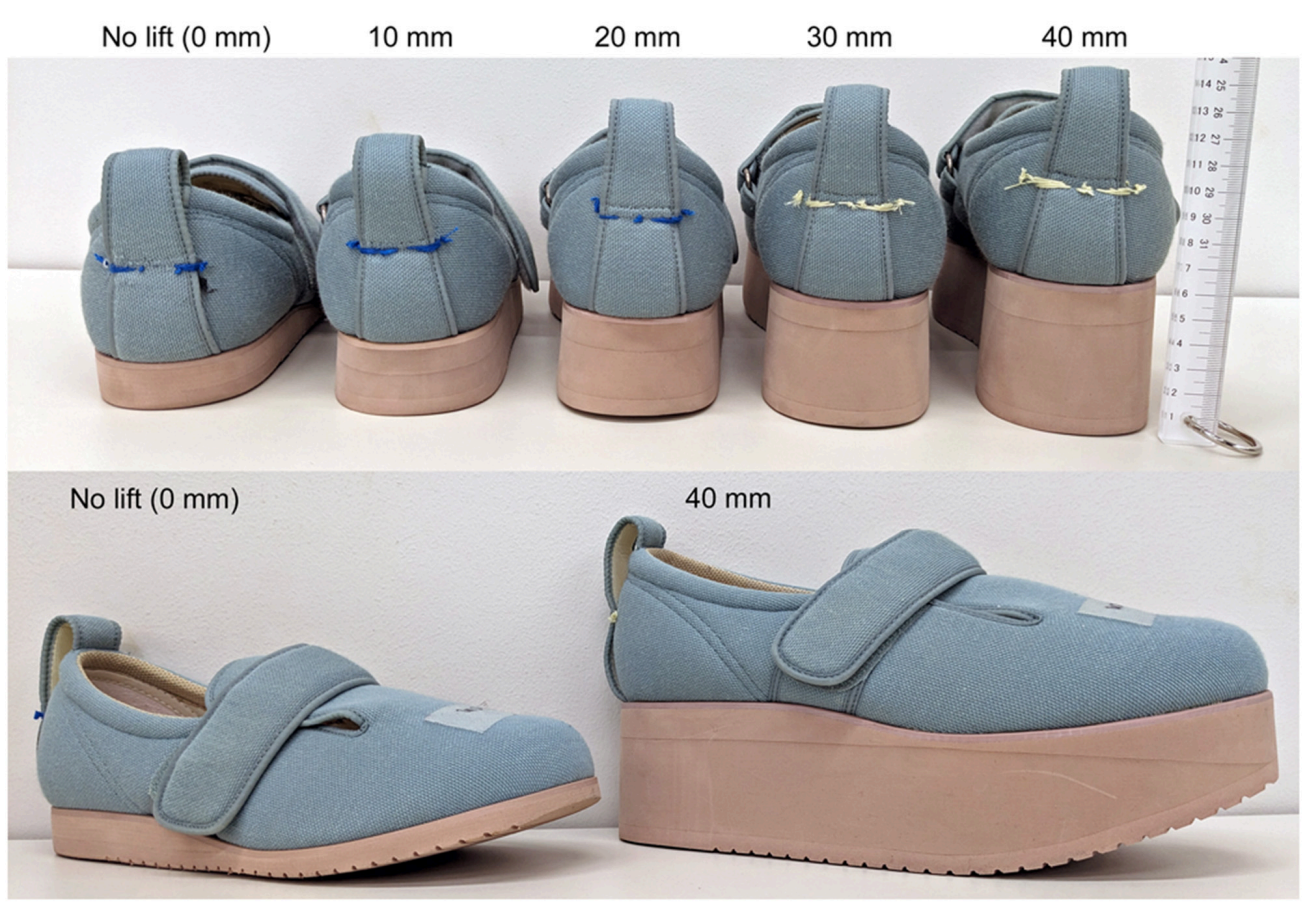
Right shoes with no lift and 10-, 20-, 30-, and 40-mm lifts. Outsole thicknesses for the 0- and 40-mm shoe lifts were 20 and 60 mm at the heel, 10 and 50 mm at the metatarsal head, and 0.8 and 32 mm at the toe, respectively.

Participants performed walking trials with no lifts and 10-, 20-, 30-, and 40-mm shoe lifts (Double Magic II, Tokutake Corp., Kagawa, Japan) in their right shoe (Figure 1). Two shoe sizes (23.0–23.5 and 27.0–27.5 cm) were used. The shoe model selected was a rehabilitation shoe with high adjustability, featuring a wide opening and a unique hook-and-loop strap system that allows for dual-point adjustment, ensuring a customized and secure fit for various foot shapes. The fit for each participant was fine-tuned using insoles, and all participants confirmed a comfortable and secure fit before the trials began.

**Figure 2:**
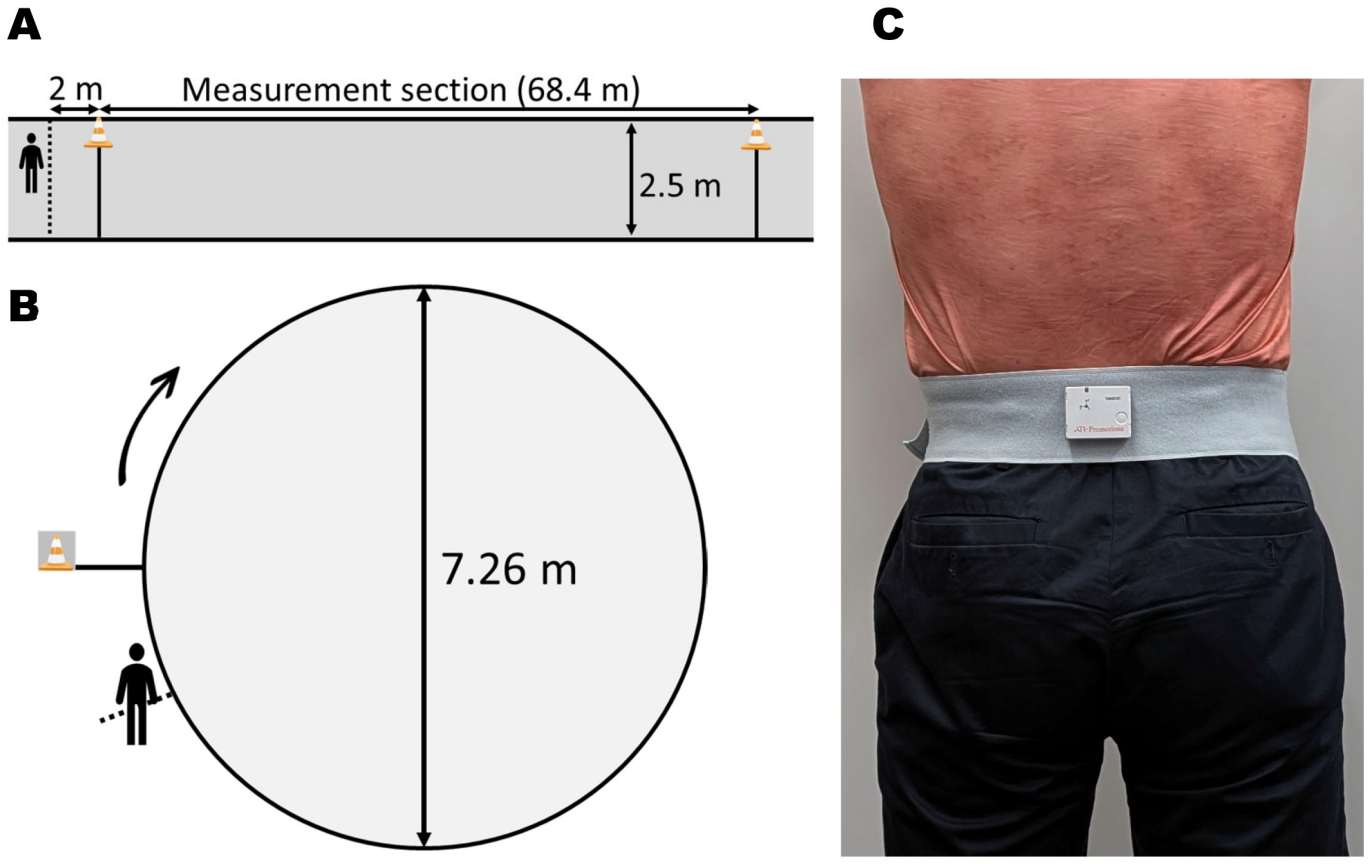
Schematic of the experimental setup. **(A)** Top-down view of the straight walking track with a 68.4-m measurement section. **(B)** Top-down view of the circular walking track (22.8-m circumference), indicating the diameter (7.26 m) and clockwise direction of walking. **(C)** Placement of the triaxial accelerometer on the third lumbar vertebra (L3).

The participants walked on a 68.4-m, flat, straight track (Figure 2A) in a university corridor during the straight walking task. This distance was set to allow the RT task to be repeated eight times, based on a previous study (Suzuki et al., 2019). During the circular walking task, the participants walked three times in a clockwise direction around a circular track with an approximate circumference of 22.8 m (Figure 2B) in a laboratory space. They were instructed to walk at their pace, starting on a 2-m path before entering the track. This combination of applying the shoe lift to the inner limb during the circular walking task was intended to create a more challenging walking task. The design was based on the hypothesis that effects of turning and LLD on gait asymmetry would be additive.

### 2.3 Procedure

The attentional demands during walking were assessed using a dual-task methodology with the RT task (Lajoie et al., 1993). Specifically, the more difficult a primary motor or cognitive task is to perform, the slower the RT tends to be for a secondary task. Participants performed the RT and walking tasks simultaneously under each of the five shoe-lift conditions. The RT was measured using a smartphone application whose accuracy and precision have been previously reported (Suzuki et al., 2016). Participants were instructed to hold the Zenfone 3 smartphone (ZE520KL, ASUSTeK Computer Inc., Taiwan, sampling interval of approximately 5 ms) in their dominant hand and dorsiflex the wrist when they felt a vibration stimulus-induced at random intervals (4000–6000 ms) from the smartphone. If the longitudinal angular velocity of the participant's response did not reach the set threshold (±4 rad/s), the attempt was excluded from the analysis. Since clarifying the primary task is necessary (Pashler, 1994), the participants were instructed to prioritize walking at a comfortable pace over the RT task.

A triaxial accelerometer (TSND151, ATR-promotions Inc., Kyoto, Japan) was used to measure step time and trunk movement during walking. Additionally, the sensor was fixed near the spinous process of each participant's third lumbar vertebra (Moe-Nilssen and Helbostad, 2002) (Figure 2C), and sampling was performed at 200 Hz. Acceleration data was processed using a low pass filter at 20 Hz and LabChart 8 software (AD Instruments, Bella Vista, NSW, Australia). The acceleration data were analyzed in increments of 15 consecutive left and right steps after the initial 20 steps of each trial. Left and right steps were identified using medial-lateral acceleration, and a foot flat on one side was identified using vertical acceleration (Auvinet et al., 2002). This marker was chosen for its distinct and reproducible signature in the lumbar accelerometer signal. Long-leg step time was then defined as the time from the foot flat of the long leg to the contralateral foot flat of the short leg. The root mean square (RMS) values of the vertical, medial-lateral, and cranial-caudal accelerations during this interval were calculated as indicators of trunk movement (Menz et al., 2003).

All included participants were permitted to practice LLD walking on a track once with each shoe lift, learned how to use the application, and practiced the RT task before the experimental trials. The order of the five shoe-lift conditions was randomized for each participant using a computer-generated random sequence. One walking task for each condition was considered as one set; in total, 15 sets (three for each of the five conditions) were performed with a short rest period between sets. The RT task was repeated eight times during each set. The walking time and step count for each set were measured from the start line to the end line by an experienced investigator. The entire procedure required approximately 40 min.

### 2.4 Statistical analysis

The mean RT for 24 trials in each condition was used in the final analysis, and data exceeding three standard deviations from the mean and standard deviation calculated for each participant within each condition were excluded from the analysis (Miller, 1991). Additionally, the mean walking time and step count were determined for each condition by averaging the values across the three sets. The cadence was computed by dividing the mean step count by the mean walking time for each condition.

The ratio of the long-leg to short-leg step times was determined from the mean step time of the short (left) and long (right) legs. Similarly, the ratios of long-leg and short-leg RMS values were calculated using the mean vertical, medial-lateral, and cranial-caudal acceleration RMS values for the short and long legs. These ratios served to represent left/right symmetry in step time and trunk movement during walking.

Two-way mixed analysis of variance was used to analyze the RTs, step-time ratios, walking time, cadence, and RMS ratios based on LLD size (0, 10, 20, 30, and 40 mm) and walking track (straight and circular tracks). Assumptions of normality (evaluated using the Shapiro–Wilk test) and sphericity were assessed. As sphericity was not satisfied, the Greenhouse–Geisser correction was applied. Following a significant main effect of LLD size, *post-hoc t*-tests with Bonferroni correction were performed. Pairwise comparisons with other LLD conditions were conducted against the no-LLD control condition to identify the degree of LLD that elicits effects in contrast to normal gait symmetry. All statistical analyses were conducted using IBM SPSS Statistics for Windows, version 30 (IBM Corp., Armonk, N.Y., USA). Statistical significance was set at *p* < 0.05.

## 3 Results

Table 1 presents the means and standard deviations for all measurement items. The results of the two-way mixed analysis of variance are presented below.

**Table 1:** Participants' measurements according to walking track and shoe lift.

**Measure**	**0 mm**	**10 mm**	**20 mm**	**30 mm**	**40 mm**
**Reaction times (ms)**					
Straight track	226.6 (21.2)	230.3 (22.8)	232.4 (21.1)	235.3 (19.8)	232.8 (22.4)
Circle track	215.1 (25.4)	220.2 (25.4)	223.7 (26.8)	220.8 (24.3)	223.1 (28.7)
Total	220.5 (23.9)	224.9 (24.3)^*^	227.7 (24.4)^*^	227.6 (23.1)^*^	227.6 (26.0)^**^
**Step-time ratio**					
Straight track	1.00 (0.03)	1.02 (0.03)	1.04 (0.04)	1.08 (0.04)	1.11 (0.04)
Circle track	1.01 (0.03)	1.04 (0.02)	1.06 (0.02)	1.10 (0.03)	1.13 (0.03)
Total	1.00 (0.03)	1.03 (0.03)^***^	1.05 (0.03)^***^	1.09 (0.04)^***^	1.12 (0.04)^***^
**Walking time (s)**					
Straight track	49.3 (6.7)	49.1 (6.4)	49.3 (7.3)	49.9 (7.6)	50.3 (7.7)
Circle track	59.1 (9.6)	59.8 (9.7)	59.8 (9.6)	60.9 (9.9)	61.7 (9.9)
Total	54.5 (9.6)	54.8 (9.8)	54.9 (10.0)	55.8 (10.4)^*^	56.4 (10.5)^***^
**Cadence (steps/s)**					
Straight track	1.94 (0.11)	1.94 (0.11)	1.92 (0.12)	1.91 (0.12)	1.90 (0.12)
Circle track	1.85 (0.16)	1.83 (0.16)	1.83 (0.16)	1.81 (0.16)	1.80 (0.15)
Total	1.89 (0.14)	1.88 (0.15)^*^	1.88 (0.15)^*^	1.86 (0.15)^***^	1.85 (0.15)^***^
**Vertical acceleration RMS ratio**					
Straight track	0.98 (0.08)	0.95 (0.08)	0.94 (0.09)	0.91 (0.12)	0.91 (0.14)
Circle track	0.93 (0.09)	0.90 (0.10)	0.88 (0.10)	0.85 (0.12)	0.84 (0.12)
Total	0.95 (0.09)	0.92 (0.10)	0.91 (0.10)^*^	0.88 (0.12)^*^	0.87 (0.13)^**^
**Medial-lateral acceleration RMS ratio**					
Straight track	0.92 (0.17)	0.84 (0.14)	0.84 (0.15)	0.76 (0.11)	0.72 (0.16)
Circle track	0.85 (0.18)	0.73 (0.16)	0.72 (0.17)	0.64 (0.18)	0.62 (0.17)
Total	0.88 (0.17)	0.78 (0.16)^***^	0.78 (0.17)^**^	0.70 (0.16)^***^	0.67 (0.17)^***^
**Cranial-caudal acceleration RMS ratio**					
Straight track	1.00 (0.05)	1.01 (0.07)	0.98 (0.10)	0.93 (0.09)	0.91 (0.14)
Circle track	1.02 (0.09)	1.02 (0.09)	0.98 (0.11)	0.95 (0.10)	0.89 (0.09)
Total	1.01 (0.07)	1.01 (0.08)	0.98 (0.10)	0.94 (0.09)^***^	0.90 (0.11)^***^

Data expressed as means (standard deviations). RMS, root mean square. Straight track group (n = 14); Circle track group (n = 16). Step-time ratio and RMS show the values of the short leg divided by the long leg. The analysis results from pairwise comparisons against the 0 mm LLD control condition are indicated by asterisks in the row showing total sample data. ^*^p < 0.05. ^**^p < 0.01. ^***^p < 0.001.

### 3.1 Reaction time

**Figure 3:**
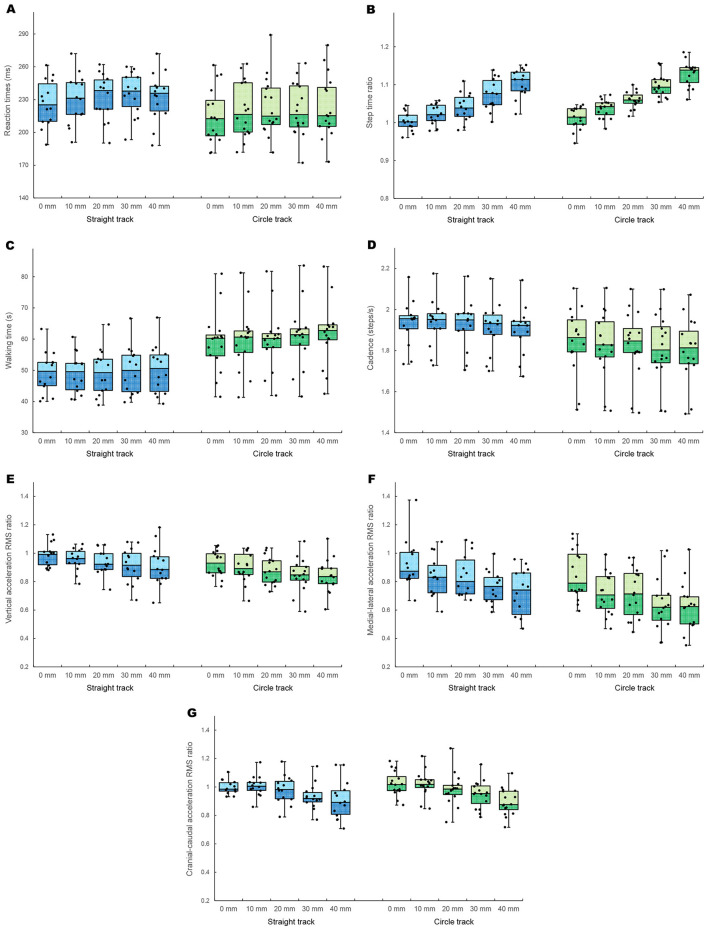
Jittered boxplot of the straight (blue) and circular (green) walking experiments for LLD size (0, 10, 20, 30, and 40 mm). Reaction time **(A)**, step-time ratio **(B)**, walking time **(C)**, cadence **(D)**, vertical acceleration RMS ratio **(E)**, medial-lateral acceleration RMS ratio **(F)**, and cranial-caudal acceleration RMS ratio **(G)**. RMS, root mean square; LLD, leg length discrepancy.

The LLD significantly affected RT (*F*_2.9, 81.8_ = 5.32; *p* = 0.002), demonstrating a large effect ($\eta_{\text{p}}^{2}$ = 0.16) (Figure 3A). However, the walking track (*F*_1, 28_ = 1.65; *p* = 0.209; $\eta_{\text{p}}^{2}\;=$ 0.06) and interaction between the LLD and walking track (*F*_2.9, 81.8_ = 0.70; *p* = 0.553; $\eta_{\text{p}}^{2}$ = 0.02) did not significantly affect RT. *Post-hoc* tests revealed that RTs were significantly longer in all LLD conditions (10, 20, 30, and 40 mm) than in the no-LLD (0 mm) condition (10 mm: *p* = 0.012; *d* = 0.18; 20 mm: *p* = 0.018; *d* = 0.30; 30 mm: *p* = 0.011; *d* = 0.30; and 40 mm: *p* = 0.009; *d* = 0.29), with small effect sizes (*d* = 0.18–0.30) (Table 1). However, the mean RT values for the 10-, 20-, 30-, and 40-mm LLD conditions did not show a systematic increase with the LLD size and remained relatively similar (Figure 3A).

### 3.2 Step-time ratio

The ratio of the long-leg to short-leg step times revealed a significant main effect of LLD size (*F*_2.0, 56.1_ = 149.77; *p* < 0.001), demonstrating a large effect ($\eta_{\text{p}}^{2}$ = 0.84) (Figure 3B). In contrast, the walking track (*F*_1, 28_ = 3.57; *p* = 0.069; $\eta_{\text{p}}^{2}$ = 0.11) and interaction effect (*F*_2.0, 56.1_ = 0.77; *p* = 0.469; $\eta_{\text{p}}^{2}$ = 0.03) were not significant. *Post-hoc* tests revealed that the step-time ratio was significantly higher in all LLD conditions than in the no-LLD (0 mm) condition (10 mm: *p* < 0.001; *d* = 0.84; 20 mm: *p* < 0.001; *d* = 1.47; 30 mm: *p* < 0.001; *d* = 2.45; and 40 mm: *p* < 0.001; *d* = 3.44), with large and increasing effect sizes (Table 1). As shown in Figure 3B, the mean step-time ratio increased incrementally as LLD size increased.

### 3.3 Walking time and cadence

LLD (*F*_1.8, 49.5_ = 14.70; *p* < 0.001) and walking track (*F*_1, 28_ = 11.52; *p* = 0.002) significantly affected walking time (Figure 3C), demonstrating large effects ($\eta_{\text{p}}^{2}=$ 0.34 and $\eta_{\text{p}}^{2}=$ 0.29, respectively). However, the interaction effect did not significantly affect walking time (*F*_1.8, 49.5_ = 2.72; *p* = 0.082; $\eta_{\text{p}}^{2}$ = 0.09). Walking time was substantially longer during circular walking than during straight walking (Figure 3C). *Post-hoc* tests for the LLD main effect revealed that the walking times were significantly longer with a 30-mm (*p* = 0.014; *d* = 0.13) and 40-mm (*p* < 0.001; *d* = 0.18) LLD than when no LLD was used, with small effect sizes (Table 1). However, the mean walking time values showed minimal systematic change across all LLD conditions (Figure 3C).

LLD significantly affected cadence (*F*_2.2, 62.0_ = 21.40; *p* < 0.001), demonstrating a large effect ($\eta_{\text{p}}^{2}$ = 0.43) (Figure 3D). In contrast, the walking track did not significantly affect cadence (*F*_1, 28_ = 3.52; *p* = 0.071; $\eta_{\text{p}}^{2}$ = 0.11), showing a medium effect size. The interaction effect was also not significant (*F*_2.2, 62.0_ = 0.83; *p* = 0.450; $\eta_{\text{p}}^{2}$ = 0.03). *Post-hoc* tests revealed that cadence was significantly lower under all LLD conditions than when no lift was used (10 mm: *p* = 0.033; *d* = 0.06; 20 mm: *p* = 0.046; *d* = 0.09; 30 mm: *p* < 0.001; *d* = 0.20; and 40 mm: *p* < 0.001; *d* = 0.28), with small effect sizes (Table 1).

### 3.4 Trunk acceleration RMS ratios

Figures 3E–G shows the results for the vertical, medial-lateral, and cranial-caudal acceleration RMS ratios of the long leg to short leg, which indicate left/right asymmetry. LLD significantly affected all three ratios (vertical: *F*_2.7, 75.8_ = 6.00; *p* = 0.001; $\eta_{\text{p}}^{2}$ = 0.18; medial-lateral: *F*_2.6, 71.9_ = 22.20; *p* < 0.001; $\eta_{\text{p}}^{2}$ = 0.44; cranial-caudal: *F*_2.2, 60.4_ = 19.46; *p* < 0.001; $\eta_{\text{p}}^{2}$ = 0.41), demonstrating large to significantly large effects. The walking track significantly affected vertical (*F*_1, 28_ = 4.34; *p* = 0.047; $\eta_{\text{p}}^{2}$ = 0.13) and medial-lateral (*F*_1, 28_ = 4.57; *p* = 0.041; $\eta_{\text{p}}^{2}$ = 0.14) ratios but not cranial-caudal ratio (*F*_1, 28_ = 0.08; *p* = 0.781; $\eta_{\text{p}}^{2}$ = 0.00). Additionally, the interaction effect was not significant for any of the three ratios (vertical: *F*_2.7, 75.8_ = 0.05; *p* = 0.980; $\eta_{\text{p}}^{2}$ = 0.00; medial-lateral: *F*_2.6, 71.9_ = 0.31; *p* = 0.786; $\eta_{\text{p}}^{2}$ = 0.01; cranial-caudal: *F*_2.2, 60.4_ = 0.51; *p* = 0.618; $\eta_{\text{p}}^{2}$ = 0.02). *Post-hoc* tests revealed that all LLD conditions were significantly lower than the no-LLD condition for all three ratios: vertical (20 mm: *p* = 0.012; *d* = 0.51; 30 mm: *p* = 0.012; *d* = 0.70; and 40 mm: *p* = 0.008; *d* = 0.72), medial-lateral (10 mm: *p* < 0.001; *d* = 0.64; 20 mm: *p* = 0.002; *d* = 0.62; 30 mm: *p* < 0.001; *d* = 1.12; and 40 mm: *p* < 0.001; *d* = 1.25), and cranial-caudal (30 mm: *p* < 0.001, *d* = 0.87; and 40 mm: *p* < 0.001, *d* = 1.16) ratios (Table 1). The walking track effect indicated greater asymmetry during circular walking for the vertical and medial-lateral ratios (Figures 3E, F).

## 4 Discussion

This study investigated the effects of LLD magnitude and walking track (straight vs. circular track) on attentional demands during walking (RT), gait temporal parameters, and trunk movement, including gait asymmetry. Our results showed that LLD significantly affected almost all measured parameters. However, the effect of the walking track was less prominent than that of LLD. No significant interaction was observed between LLD and walking track for any parameter, suggesting that the effects of both factors were largely independent.

### 4.1 Attentional demands

The primary finding of this study was that RT during walking was significantly delayed even with an LLD of just 10 mm. The LLD factor had a statistically robust impact on RT, with a large overall effect size. However, it is important to interpret the practical significance of this effect with caution. Although an established minimal clinically important difference for this task was not available, the absolute magnitude of the RT delay observed in this study did not exceed 10 ms, and the pairwise effect sizes (Cohen's d) comparing LLD conditions to baseline were small. This observation suggests that, although the presence of an LLD increases the complexity of motor control, which in turn acts as a trigger for greater attentional demands, the extent of this increased load was relatively limited and smaller than that observed in our previous study (Suzuki et al., 2019). This finding, however, should be contextualized by the current study's focus on young, healthy participants adapting to an acute perturbation.

We hypothesized that increased attentional demands would be greater when the asymmetric circular track rather than the symmetric straight track was used. However, this did not occur in this study, and no interaction between the effects of the walking path and those of greater LLDs was detected. Attentional demands were affected by gait asymmetry due to LLD but not the walking track, suggesting that combining these asymmetric elements does not further increase attentional demands. Therefore, as was also evident in other parameters, the circular track defined in this study did not contribute to interaction effects.

### 4.2 Gait temporal parameters

The simulated LLD created an asymmetry in step timing between the short and long legs, increasing the step-time ratio. Additionally, the step-time asymmetry became more pronounced as LLD increased. However, the type of walking track did not significantly affect the step-time ratio. Uncorrected LLD is associated with a decreased short-leg step time (Bhave et al., 1999) and leads to an earlier push-off in the short leg (Perttunen et al., 2004). A decreased clearance of the long leg would result in the earlier initial contact of the long leg. LLD also increases the vertical displacement of the center of gravity (COG) (Gurney, 2002), which increases during the first half of the long-leg stance phase and drops significantly in the second half due to the delayed initial contact of the short leg. This pattern of step-time alteration, where short-leg step time decreased and long-leg step time increased, was observed in the present study.

Walking time was significantly affected by LLD and walking track. It was substantially longer on the circular track than on the straight track and further increased when LLD exceeded 30 mm. The longer walking time on the circular track corresponds to a decrease in walking speed. Notably, walking speed is determined by cadence and stride length (Murray et al., 1966), which are regulated separately (Ardestani et al., 2016). Given that cadence did not significantly change on the circular track (as shown in the Results), this walking speed reduction is likely attributed to a decrease in stride length during circular walking.

Cadence was significantly affected by LLD, but not the walking track. The lack of a walking track effect, contrasting with speed changes, suggests that circular adaptations primarily involve stride length rather than stepping rate. Additionally, the decrease in cadence with increasing LLD is likely related to increased step-time asymmetry, making symmetrical rhythm challenging and reducing stepping frequency. This LLD-induced reduction in cadence likely represents a cautious gait strategy, which is a top-down adaptation by the central nervous system to ensure stability in response to the novel asymmetric perturbation.

### 4.3 Trunk movement

Accelerations in the vertical, medial-lateral, and cranial-caudal directions in the lumbar region, close to the COG, change similarly and repetitively between the left and right steps during normal gait (Auvinet et al., 2002). The present study results indicate that the symmetrical trunk movements in the vertical and medial-lateral directions were asymmetric due to LLD and the circular track. However, no interaction between the LLD and the track type was detected, and the asymmetry resulting from walking on the circular track was generally similar to that due to an LLD of 20 mm. An LLD increases the vertical displacement of the COG (Gurney et al., 2001) since the pelvis on the short-leg side drops significantly during the long-leg stance phase (Kaufman et al., 1996). Furthermore, Pereiro-Buceta et al. (2021) reported that even small LLDs decrease the load applied during the long leg stance phase, suggesting that increasing LLD enhances the asymmetry of the gait pattern. Although no change in the medial-lateral displacement of the body's COG due to LLD has been reported, it presumably deviates to the short-leg side. Consequently, the COG moves more laterally and downward, generating larger acceleration toward the short leg stance phase than toward the long leg stance phase. This acceleration would make the motor control needed for deceleration in the early stance phase of the short leg more difficult. Given that the short leg was positioned outside of the circular track in the present study, the body's COG was likely compelled to deviate further laterally due to the centrifugal force during the stance phase. Regarding the cranial-caudal direction, the RMS ratio was unaffected by the walking track—likely because the control of turning primarily challenges the medial-lateral plane, while the concurrent reduction in walking speed diminished the magnitude of accelerations in the propulsive direction. However, the RMS ratio decreased with increasing LLD, indicating relatively greater trunk acceleration RMS during the short-leg stance phase than during the long-leg stance phase when a moderate LLD was simulated.

### 4.4 Gait asymmetry on adaptation and attentional demands

Functional compensation for an LLD during walking can be achieved through several methods (Gurney, 2002; Hoikka et al., 1989; Kaufman et al., 1996; Perttunen et al., 2004; Walsh et al., 2000). Since mild LLD affects leg biomechanics during walking (Khamis and Carmeli, 2018), the body is required to adapt to any minor asymmetries. Findings from trunk movement analysis indicated that asymmetry occurred under mild LLD conditions on the circular track. However, the circular track had a radius of approximately 3.6 m, which did not inhibit step-time symmetry or reduce cadence during walking. It also did not increase the attentional demands. Walking on a curved path is a common activity to which participants may have adapted and was therefore likely an insufficiently challenging task. In contrast, the LLD condition used in this study was simulated using shoe lifts, and the lift height was changed randomly during the experiment, resulting in conditions that were difficult to adapt to.

The step-time results indicate that the alternating movements of the left and right legs during walking become asymmetric when shoe lifts are used to create an LLD. Alterations in the load and joint movements during walking due to the artificial lift may create bilateral differences in the afferent sensory input to the central pattern generator, presenting a significant challenge to automatic gait control. This increased task difficulty may necessitate greater involvement of higher centers, consistent with findings indicating that increased task difficulty alters brain activity in gait control (Hamacher et al., 2015). Therefore, the increased motor-control demand of responding to these bilateral challenges imposed by LLD likely necessitated greater higher-center involvement, resulting in increased attentional demands during asymmetrical walking than during symmetrical walking. Similar to how instability during walking increases attentional demands (Suzuki et al., 2019), the asymmetry of trunk movements due to LLD may also increase attentional demands, likely because it makes postural control during walking difficult, particularly in the short leg stance phase. Walking speeds are typically slower when a moderate shoe lift is used or when walking on a circular track. However, decreased walking speed in treadmill walking does not affect the attentional demands (Abernethy et al., 2002). Therefore, the task characteristics and physical functions causing decreases in walking speed, rather than that reduction, are likely the important factors affecting attentional demands.

### 4.5 General discussion

This study investigated the effects of increasing simulated LLD and different walking conditions on the gait of young, healthy participants. The instantaneous control of asymmetry in the legs increases the attentional demands during walking. However, in patients with acquired LLD, the LLD may initially be compensated for when the difference is gradually increased, followed by the acquisition of an adaptive gait. If the temporal elements of symmetry can be maintained by compensation, attentional demands may not increase. Although this type of compensation has the advantage of preserving symmetry, it may lead to secondary symptoms, such as back pain and joint deformity (Vogt et al., 2020). Furthermore, the asymmetry caused by the walking path did not affect the attention demands. This study's participants were already proficient at circular walking, indicating that the motor control task had strong elements of stability and predictability.

In addition to this primary limitation, our study has several other methodological aspects that warrant discussion. Our study did not include a quantitative assessment of participants' individual biomechanical characteristics, such as musculoskeletal condition or leg dominance. This choice, along with our focus on temporal rather than spatial gait parameters, was a consequence of this study's primary objective to assess attentional demands during a continuous, long-distance walking task to ensure ecological validity. Nevertheless, the step-time ratio in the baseline (0 mm LLD) condition was approximately 1.00, indicating that the participant group exhibited functionally symmetrical gait.

The increase in attentional demands observed in young, healthy participants in this study was small. This may be because healthy adults are capable of adaptive gait control, as healthy males have been shown to retain lateral stability through compensatory motor control under a 30-mm LLD condition (Miyagi et al., 2023). In contrast, Gurney (2002) suggested that older adults find it more difficult to adapt to a larger LLD than younger individuals, indicating that LLD has a greater impact on attentional demands in individuals with reduced physical functions. Future studies should consider the effects of LLD on motor and attention functions. Even if gait asymmetry due to LLD becomes a factor in increasing attentional demands, clarifying the extent to which functional compensation can mitigate this effect is necessary. Furthermore, investigating how attentional demands in patients with and without functional compensation for LLD vary when using shoe lifts or insoles to maintain gait symmetry is necessary. These findings will help clarify the effects of therapeutic interventions in terms of attention functions.

In conclusion, this study examined the impact of simulated LLD and walking track on attentional demands and gait symmetry in young, healthy adults. Simulated LLD significantly increased attentional demands and gait asymmetry, although the increase in attentional demands was limited in healthy participants. The circular walking track had minimal effects and did not interact with LLD. These results provide insight into the effects of gait asymmetry caused by the degree of LLD and walking environment on human gait strategy and its associated attentional demands.

## Data Availability

The raw data supporting the conclusions of this article will be made available by the authors, without undue reservation.
